# Temporal context shapes multicue integration in auditory time-to-contact judgments

**DOI:** 10.1038/s41598-026-61087-4

**Published:** 2026-07-13

**Authors:** Alice Bollini, Claudio Campus, Melis Ince, Luigi F. Cuturi, Monica Gori

**Affiliations:** 1 Unit for Visually Impaired People, Istituto Italiano di Tecnologia, Genoa, Italy; 2 NeuroBRITE Research center, Italian Multiple Sclerosis Foundation (FISM), Genoa, Italy; 3 Department of Psychology, Humboldt-Universität zu Berlin, 10099 Berlin, Germany; 4 Berlin School of Mind and Brain, Humboldt-Universität zu Berlin, 10117 Berlin, Germany; 5 Department of Cognitive Sciences, Psychology, Education, and Cultural Studies, University of Messina, Messina, Italy; 6 HIMC, Florida Institute for Human and Machine Cognition, Pensacola, FL USA

**Keywords:** Time-to-contact (TTC), Auditory perception, Multicue integration, Bayesian inference, Temporal context, Predictive coding, Neuroscience, Psychology, Psychology

## Abstract

Anticipating when a moving object will arrive is fundamental for action. While vision-based time-to-contact (TTC) judgment follows reliability-weighted integration of motion and duration cues, it remains unclear whether audition supports similar computations. We asked listeners to judge TTC for moving sounds in motion-only, duration-only, and combined cue conditions, under fast and slow temporal contexts. Combined cues consistently improved precision, but temporal context reshaped bias. Indeed, integration generates remarkable delays in the fast regime and reduced anticipatory errors in the slow regime. Regression-to-the-mean effects revealed strong influences of context-dependent priors. A hierarchical model further suggested that adjusted cue weights in a manner broadly consistent with reliability-based cue use, while deviations from the flat-prior benchmark indicated that temporal context and priors shaped performance. These results indicate that auditory TTC is not a fixed computation, but an adaptive inference process shaped by temporal context, revealing a modality-specific route to predictive timing.

## Introduction

 The ability to anticipate the arrival of an approaching object is critical for survival and effective action. Accurate time-to-contact (TTC) estimation allows humans to interact effectively with a dynamic world, from braking at a traffic light to intercepting a ball. Classically, TTC perception has been explained by the *tau* (τ) variable, which specifies time-to-collision based on optical expansion^1,2^. However, this account does not fully explain human TTC judgments across more complex situations. Observers frequently show systematic errors, especially under acceleration, non-collision trajectories, or conditions in which optical expansion is ambiguous or unavailable^3,4^. These deviations suggest that TTC estimation is not based solely on a direct readout of optical tau but also on additional information sources, such as perceived distance, velocity, elapsed time, and contextual expectations.

A central question is how the brain combines different types of information to estimate TTC. One distinction is between *kinematic cues* (e.g., velocity, distance; TTC = d/v) and *temporal cues* (e.g., elapsed duration or interval timing). Although mathematically interdependent, these cues are represented and processed differently in the brain^5,6^. Vision research shows that humans integrate cues in a reliability-weighted fashion, approximating Bayesian optimality^7,8^, including duration and motion cues for TTC estimation^9^. These findings support the broader view of the brain as a Bayesian inference machine^10,11^, where the combination of noisy sensory inputs and prior expectations shapes perception.

Audition provides a robust test case for these theories. While auditory motion processing is relatively coarse in both position and velocity domains—for example, velocity discrimination thresholds for moving sounds are around 5–15°/s when spatial information is limited, improving to ~ 3–7°/s with displacement cues^12,13^; in contrast, auditory temporal processing supports discrimination of intervals and durations with thresholds in the order of only a few milliseconds (e.g., gap detection thresholds of ~ 2–5 ms in normal-hearing listeners;^14,15^. This asymmetry suggests that auditory TTC judgments may rely more strongly on temporal anchoring (duration-based estimation) than kinematic extrapolation (velocity-based projection). Previous work has confirmed that auditory TTC estimation is possible^16–19^, but less is known about whether and how auditory cues are integrated optimally, or the extent to which temporal context modulates their use.

Temporal context may be a critical factor in TTC estimation. By temporal context, we refer to the characteristic timescale of the task, that is, the distribution of intervals within an experimental session that calibrates current timing behavior. In Bayesian terms, this context serves as an induced temporal prior, learned from recent interval history, and shapes how incoming sensory evidence about duration or motion is weighted when estimating time-to-contact.Jazayeri and Shadlen^20^, showed that interval timing is calibrated by temporal context, with priors exerting a stronger influence under uncertainty. Related interval-timing work further shows that humans can learn internal representations of temporal statistics and that duration judgments can exhibit central-tendency effects toward recently experienced intervals^21,22^. Together, these findings raise the possibility that TTC judgments may also operate as adaptive inferences shaped by temporal context. In our design, fast sessions (1–2 s TTCs) created a short temporal context, whereas slow sessions (3–6 s TTCs) established a longer one. Based on the principle that uncertainty increases prior influence^20^ and on the relatively coarse nature of auditory motion processing^12^, we predicted that fast contexts would reduce the relative reliability of motion-derived TTC estimates, leading to lower relative weighting of motion information. By contrast, slow contexts may allow a more balanced contribution of motion- and duration-based information.

The present study directly tests whether temporal context modulates auditory cue integration by extending the Prediction Motion paradigm^9^ to the auditory domain. Using a loudspeaker array, we simulated frontoparallel motion under three conditions: motion-only (AM), duration-only (AD), and combined (AMD). Contrasting fast and slow sessions, we asked whether listeners combine temporal and kinematic cues in a reliability-weighted manner, whether integration strategies shift with temporal context, and how priors shape auditory TTC judgments. This study offers a critical test of how the brain flexibly constructs time by linking a fundamental perceptual task to broader theories of Bayesian inference and predictive coding.

## Method

### Sample

Fifteen healthy volunteers (8 female; mean age = 29 years, range 24–37) participated in the study. Fourteen were right-handed, and one was left-handed. All reported normal hearing. The experiment was approved by the Ethics Committee of the ASL3 Genovese (Italy); participants provided written informed consent in accordance with the Declaration of Helsinki. All methods were performed in accordance with relevant guidelines and regulations. An a priori power analysis using G*Power 3.1^23^ determined the minimum number of participants required to detect a condition effect (3 levels) in a within-subjects ANOVA design. Based on the robust effects reported by Chang and Jazayeri^9^, for similar TTC estimation tasks, we adopted a conservative medium-large effect size (η²ₚ = 0.15, corresponding to f = 0.42). With a significance level α = 0.05 and a desired power of 1 - β = 0.80, the analysis indicated that 11 participants would be sufficient. Our sample (*n* = 15) exceeded this threshold, ensuring sufficient statistical sensitivity to test the main hypothesis.

### Design

**Fig. 1:**
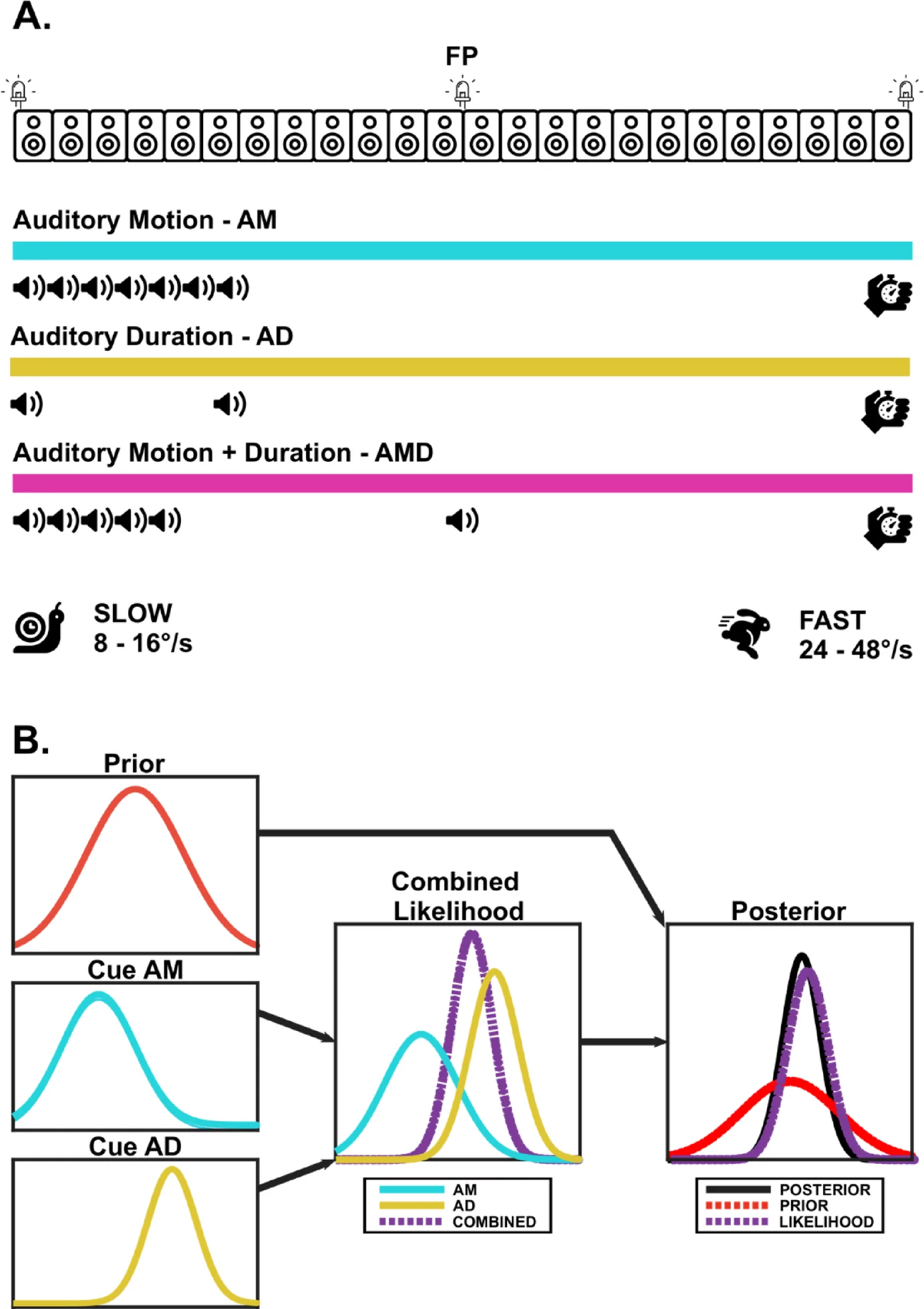
Experimental design and Bayesian cue-integration framework (**A**) Schematic of the auditory time-to-contact (TTC) task in the three conditions: auditory motion (AM), auditory duration (AD), and auditory motion + duration (AMD), shown for the FAST and SLOW temporal contexts. (**B**) Conceptual Bayesian framework. Likelihoods derived from AM and AD information are first combined into a single sensory likelihood based on their relative reliability. This combined likelihood is then integrated with the prior, which reflects expectations shaped by the task’s temporal statistics, to generate the posterior estimate of TTC.

Auditory stimuli were presented through an array of 24 loudspeakers arranged horizontally over a total length of 180 cm, corresponding to approximately 48° of visual angle when viewed from the participant’s position (202 cm from the array center). Three LEDs placed above the loudspeakers served as reference points: the lateral LEDs indicated the array’s spatial extent, while the central LED functioned as a fixation point (FP). Participants were instructed to keep their heads still and maintain central fixation throughout the experiment (Fig. 1). The experiment was conducted in a quiet, dark room to minimize distractions.

The experimental design was based on an auditory adaptation of the three timing tasks originally developed by Chang and Jazayeri^9^ in the visual domain. The goal was to engage participants in using different perceptual strategies, based on motion, duration, or motion-duration integration, to estimate time-to-contact (TTC). Stimuli consisted of temporally filtered white noise, spatialized across the speaker array (Fig. 1).

Each participant completed two experimental sessions on separate days, with order counterbalanced across participants to control session-related order effects. Sessions covered the same spatial range but differed in temporal context: In the FAST session, TTCs were short (1–2 s) and used fast speeds (~ 24–48°/s); in the SLOW session, TTCs were long (3–6 s) and used proportionally slower speeds (~ 8–16°/s). In both sessions, participants completed the following three tasks:

- **Auditory-motion (AM)**: A continuous sound moved from the start position (leftmost speaker) toward the FP and disappeared before arrival. Participants extrapolated TTC based on the perceived velocity of the sound (kinematic strategy) during the audible motion phase.
- **Auditory-duration (AD)**: Two brief (100 ms) sounds were played at the start position and another one at a midpoint along the trajectory. Participants estimated TTC based on the elapsed duration and implied motion (temporal strategy).
- **Auditory-motion + Duration (AMD)**: The stimulus started moving as in the AM condition, disappeared before reaching the FP, and then briefly reappeared (100 ms) at the FP. This condition provided both kinematic and temporal cues, allowing their integration.

Each task was presented in a separate block, and the order of tasks was counterbalanced across participants within each session. For each condition, five stimulus speeds and five auditory endpoint positions were used. Endpoint positions were identical across the FAST and SLOW sessions: 45, 60, 75, 82.5, and 90 cm along the loudspeaker array, corresponding approximately to 12°, 16°, 20°, 22°, and 24° of visual angle. The FAST session used speeds of 90, 112.5, 135, 157.5, and 180 cm/s, corresponding approximately to 24°, 30°, 36°, 42°, and 48°/s and generating actual TTCs of 2.00, 1.60, 1.33, 1.14, and 1.00 s, respectively. The SLOW session used speeds of 30, 37.5, 45, 52.5, and 60 cm/s, corresponding approximately to 8°, 10°, 12°, 14°, and 16°/s and generating actual TTCs of 6.00, 4.80, 4.00, 3.43, and 3.00 s, respectively. On every trial, the sound moved from left to right, disappeared at a certain point, and participants had to press a button when they estimated the sound would have reached the target. The target position corresponded to the end of the loudspeaker array. However, in all conditions, the auditory stimulus never reached this position. Instead, the sound was only presented up to an intermediate location (the fixation point, FP), and participants were required to estimate the time at which the sound would have reached the final target position. After each response, participants received auditory feedback indicating whether the response was early, late, or close to the actual TTC. Feedback was based on a tolerance window around the actual TTC: responses outside the window were labeled as early or late, whereas responses within the window were labeled as correct. The tolerance window was ± 250 ms in the FAST session and ± 450 ms in the SLOW session. These feedback categories were used only online to support task engagement; all behavioral analyses were based on continuous signed response errors relative to the actual TTC. Inter-trial intervals were randomized between 1.2 and 2.5 s. Importantly, because the auditory stimulus never reached the final target position and the FP served as the last available sensory event, the information available in AM, AD, and AMD conditions was not perfectly matched. Therefore, the AMD condition should be interpreted as providing combined motion and temporal information within the task structure, rather than as a strict sum of independent sensory likelihoods.

### Statistical analysis

All analyses were performed in MATLAB R2024b. The data were analyzed through a range of classical and computational approaches to characterize the use of temporal (duration), kinematic (velocity) cues, and their integration into time-to-contact (TTC) estimation. We adopted a multilevel analysis comprising:

### ANOVA on bias and precision

To assess whether the accuracy and precision of TTC estimation varied between the three conditions (AD - duration, AM - motion, AMD - integration), we conducted a repeated-measures ANOVA on the averaged data per subject. To assess the accuracy and reliability of participants’ TTC estimates, we used the bias = TTC_estimated_ - TTC_real_ as metrics, with positive values indicating overestimation and negative values indicating underestimation. Precision was defined as the within-participant standard deviation of single-trial signed errors for each condition, indexing the dispersion of TTC estimates around the physical target. Lower values indicate higher response precision.

### Flat-prior Bayesian cue-combination benchmark

To assess whether performance in the combined motion-duration condition (AMD) was consistent with reliability-weighted cue combination, we computed a benchmark prediction based on inverse-variance weighting of the single-cue estimates, following classical cue-combination frameworks^24,7^. This formulation is equivalent to maximum-likelihood integration of independent Gaussian estimates and can also be interpreted as Bayesian inference under a flat prior. Because no explicit prior is included in this benchmark, we distinguish it from the hierarchical Bayesian model described below, in which prior parameters are estimated directly. Importantly, the AM and AD variabilities used in this benchmark were empirical behavioral measures of single-cue performance. They should therefore be interpreted as observed single-cue reliabilities, rather than as pure estimates of sensory noise, because they may already include the influence of contextual priors and response strategies.

- Predicted bias:

1 $$\:\begin{array}{*{20}c} {\hat{B}_{{AMD}} = w_{{AM\:}} \bullet \:B_{{AM}} + w_{{AD}} \bullet \:B_{{AD}} \:\:\:with\:w_{i} \: = \frac{{1/\sigma \:_{i}^{2} }}{{1/\sigma \:_{{AM}}^{2} + 1/\sigma \:_{{AD}}^{2} \:}}} \\ \end{array}$$

- Predicted variability:

2 $$\:\begin{array}{*{20}c} {\sigma \:_{{AMD}} = \sqrt {\left( {\frac{1}{{1/\sigma \:_{{AM}}^{2} + 1/\sigma \:_{{AD}}^{2} }}} \right)} } \\ \end{array}$$

Predicted AMD bias and precision were compared with observed AMD performance using paired-samples t-tests. Because AM and AD precision values were derived from behavior, they were interpreted as empirical measures of observed single-cue reliability rather than as pure estimates of sensory noise. This analysis was therefore used as a descriptive flat-prior reliability-weighted benchmark for cue combination.

### Regression to the mean analysis

Following Chang and Jazayeri^9^, we assessed regression-to-the-mean effects by regressing estimated TTC against real TTC separately for each condition and temporal context. The estimated TTC was computed as the sum of the real TTC and the signed response error. Under perfect performance, the relationship between estimated and real TTC would have a slope of 1. Slopes significantly below unity indicate compression toward the mean TTC, reflecting overestimation of shorter TTCs and underestimation of longer TTCs.

### Hierarchical Bayesian model

To characterize how participants integrated prior knowledge with sensory evidence across three conditions—motion only (AM), duration only (AD), and multisensory (AMD), we analyzed the *fast* and *slow* sessions separately. For each participant, the model estimated motion- and duration-cue uncertainty (σ_AM_, σ_AD_), the prior mean and uncertainty (µ_prior_, σ_prior_), and the motion weight in multisensory trials (* w*). On each trial, the TTC estimate was treated as the maximum a posteriori (MAP) combination of sensory evidence and prior expectations. The prior parameters were treated as subjective, estimated from participants’ responses rather than fixed to the objective stimulus distribution. This choice allowed the model to capture possible differences between the experimentally defined TTC distribution and the temporal context internalized by each participant. The objective FAST and SLOW TTC distributions defined the experimental context, but we did not assume that participants represented these distributions exactly or as a fixed discrete prior. The MAP prediction is:

3 $$\:\begin{array}{*{20}c} {T\widehat{{TC}}_{{MAP}} = \frac{{\frac{1}{{\sigma \:_{{sensory}}^{2} }} \bullet \:\:TTC_{{real\:}} + \frac{1}{{\sigma \:_{{prior}}^{2} }} \bullet \:\:\mu \:_{{prior}} }}{{\frac{1}{{\sigma \:_{{sensory}}^{2} }} + \frac{1}{{\sigma \:_{{prior}}^{2} }}}}} \\ \end{array}$$

For each subject, sensory variance in the AMD condition was computed as a weighted combination of AM and AD cue variances:

4 $$\:\begin{array}{*{20}c} {\sigma \:_{{AMD1}}^{2} = w^{2} \bullet \:\sigma \:_{{AM}}^{2} \: + \left( {1 - w} \right)^{2} \bullet \:\sigma \:_{{AD}}^{2} } \\ \end{array}$$

where *w* represents the weight assigned to the movement cue (AM) and is estimated individually for each subject. This parameter was intentionally left free to quantify participants’ empirical cue-weighting behavior and to allow deviations from reliability-based optimal weighting.

To provide a reliability-based benchmark within the hierarchical model, we also computed an optimal motion weight, w_optimal_, from the model-estimated sensory uncertainties for the motion and duration cues:

5 $$\:\begin{array}{*{20}c} {w_{{optimal}} = \frac{{{\raise0.7ex\hbox{$1$} \!\mathord{\left/ {\vphantom {1 {\sigma _{{AM}}^{2} }}}\right.\kern-\nulldelimiterspace} \!\lower0.7ex\hbox{${\sigma _{{AM}}^{2} }$}}}}{{\left( {{\raise0.7ex\hbox{$1$} \!\mathord{\left/ {\vphantom {1 {\sigma _{{AM}}^{2} }}}\right.\kern-\nulldelimiterspace} \!\lower0.7ex\hbox{${\sigma _{{AM}}^{2} }$}}} \right) + ~\left( {{\raise0.7ex\hbox{$1$} \!\mathord{\left/ {\vphantom {1 {\sigma _{{AD}}^{2} }}}\right.\kern-\nulldelimiterspace} \!\lower0.7ex\hbox{${\sigma _{{AD}}^{2} }$}}} \right)}}} \\ \end{array}$$

where σ_AM_ and σ_AD_ are the model-estimated uncertainties for the motion and duration cues, respectively. Importantly, w_optimal_ was derived from the sensory uncertainty parameters estimated by the hierarchical model, not from the behavioral precision values used in the flat-prior Bayesian cue-combination benchmark, and it did not include the prior variance σ_prior_. Thus, w_AM_ was freely estimated from AMD responses, whereas w_optimal_ was used only as a reliability-based benchmark for comparison.

Trial-wise responses were modeled as Gaussian noise centered on the MAP prediction, with residual response variability scaled by the condition-specific sensory uncertainty:

6 $$\:\begin{array}{*{20}c} {\log p\:\left( {data\:|\:\theta \:} \right) = \sum \: _{i} \log N\:\left( {TTC\:_{{est}}^{{\left( i \right)}} |\:\widehat{{TTC}}_{{MAP}}^{{\left( i \right)}} ,\:\sigma \:_{{sensory}} } \right)} \\ \end{array}$$

Subject-wise initialization values were derived empirically from the observed means and standard deviations of the prior, motion-cue uncertainty, duration-cue uncertainty, and cue-weight parameters. The cue-weight parameter was initialized at 0.5, and small random jitter was added to ensure positivity. A hierarchical Bayesian model was fit separately for FAST and SLOW sessions using MATLAB’s slice sampler (5,000 iterations; burn-in 1,000; thinning 3)^25,26^. Single-chain posterior samples were obtained for each parameter and participant. Convergence and sampling error were evaluated using effective sample size (ESS), Monte Carlo standard error (MCSE), and Geweke’s z.

This procedure had two main objectives: first, to estimate cue uncertainty and the strength of the subjective prior; and second, to quantify participants’ empirical cue-weighting behavior relative to a reliability-based benchmark. We tested cue reliability differences with paired-samples t-tests on $\:{\sigma\:}_{AM}\:vs\:{\sigma\:}_{AD}$, and compared observed vs. optimal weights using paired t-tests and Pearson correlations.

Finally, we validated the model by calculating predictions for the AMD condition and comparing them with the observed responses, obtaining a value of R^2^ that quantifies how much of the variance in the data is explained by the model. This step allows us to evaluate the goodness of fit and the consistency of the behavior with a Bayesian process.

## Results

The Results section proceeds in four steps. Bias and response variability are first analyzed across task conditions and temporal contexts. Observed performance in the combined motion–duration condition (AMD) is then compared with predictions from a flat-prior Bayesian cue-combination benchmark. Regression-to-the-mean effects are subsequently examined to assess whether TTC estimates were shaped by the temporal statistics of each context. Finally, a hierarchical model is used to estimate cue uncertainty, prior parameters, and cue weighting.

### ANOVA on bias and precision

#### FAST session (short TTCs, 1–2 s)

##### Bias

**Fig. 2:**
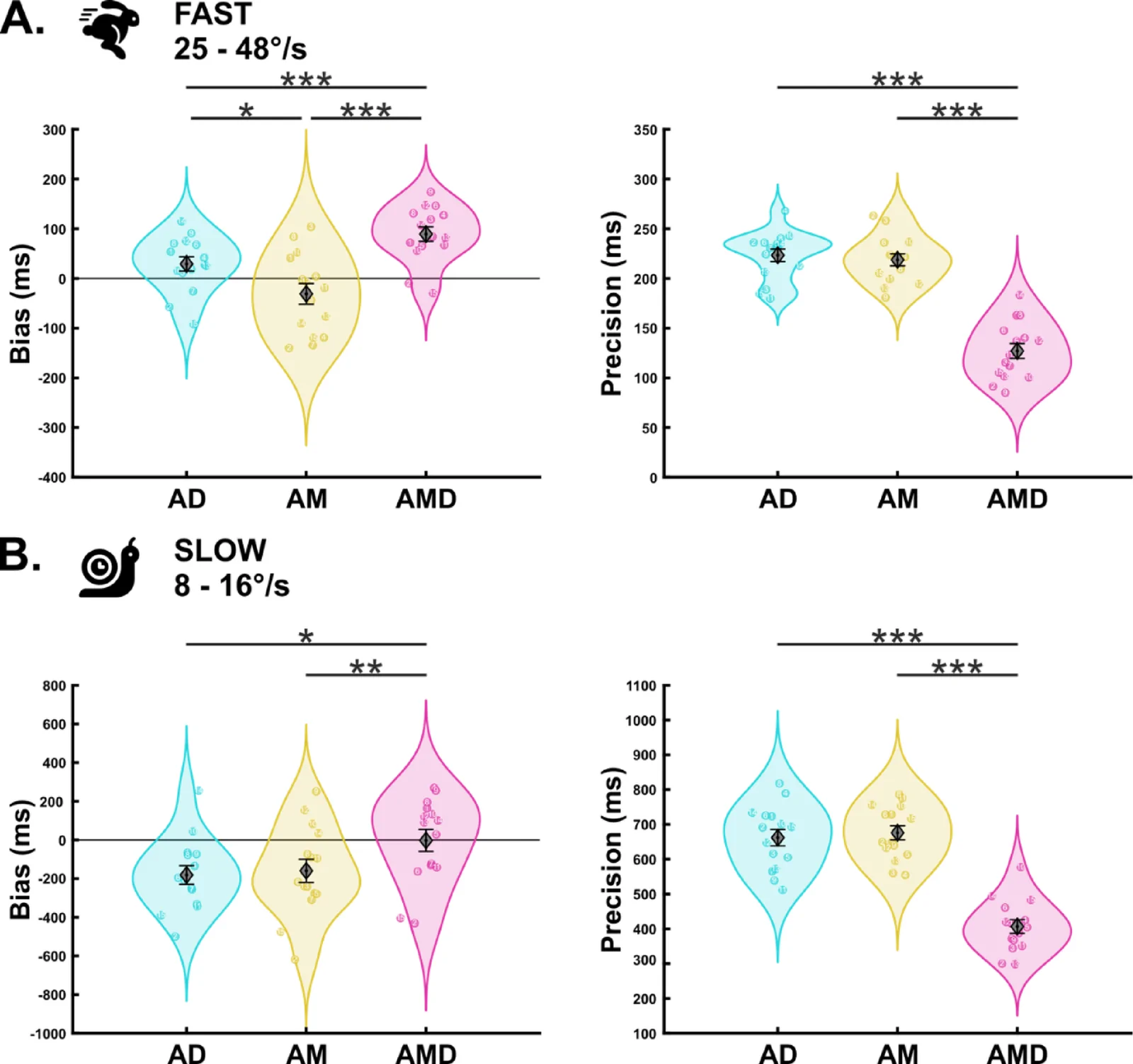
Behavioral performance across sensory conditions. (**A**) FAST (24–48°/s) and (**B**) SLOW (8–16°/s) conditions. Violin plots show individual participants’ bias (left) and precision (right) in the auditory duration (AD), auditory motion (AM), and combined motion-duration (AMD) conditions, averaged across trials within each speed regime. Each circle represents one participant, diamonds indicate group means, and error bars denote the standard error of the mean. Repeated-measures ANOVAs revealed significant effects of condition for both bias and precision (*p* < 0.01); post-hoc comparisons (Bonferroni-corrected) showed significant differences between AMD and the unimodal conditions (*p<0.5, **p < 0.1, ***p < 0.01).

The repeated-measures ANOVA revealed a significant main effect of condition on bias, F(1.26, 17.59) = 22.94, *p* < .001, η^2^ p = 0.62. Descriptive statistics showed that AMD produced the largest positive bias (M = 89.23 ms, SD = 56.47), followed by AD (M = 29.20 ms, SD = 55.46), whereas AM showed a small negative bias (M = -31.01 ms, SD = 80.30). Bonferroni-corrected post hoc comparisons showed that AD differed significantly from AM (*p* = 0.28) and from AMD (*p* < 0.01), and that AM also differed significantly from AMD (*p* < 0.01) (Fig. 2a).

##### Precision

The ANOVA on precision also revealed a significant main effect, *F*(1.79, 25.02) = 81.95, *p* < .001, η^2^ p = 0.85. AMD was the most precise (*M* = 92, *SD* = 22), while AD (*M* = 160, *SD* = 18) and AM (*M* = 157, *SD* = 17) were less precise. Post hoc comparisons showed AMD differed significantly from both AD, *t*(14) = 11.31, *p* < 0.01, *d* = 3.62, and AM, *t*(14) = 10.85, *p* < 0.01, *d* = 3.47, whereas AD and AM did not differ, *t*(14) = 0.46, *p* = 1.00 (Fig. 2b).

### SLOW session (long TTCs, 3–6 s)

#### Bias

The ANOVA on bias showed a significant main effect of condition, *F*(2, 28) = 9.57, *p* < .001, η^2^ p = 0.41. Descriptive statistics indicated that AMD exhibited the smallest bias (*M* = -3, *SD* = 221), whereas AD (*M* = − 181, *SD* = 186) and AM (*M* = -160, *SD* = 233) showed negative biases. Post hoc tests confirmed that AMD differed significantly from AD, *t*(14) = − 4.01, *p* = 0.01, *d* = –0.83, and from AM, *t*(14) = − 3.52, *p* = 0.04, *d* = –0.73. AD and AM did not differ, *t*(14) = − 0.48, *p* = 1.00, (Fig. 2c).

#### Precision

The ANOVA on precision again revealed a robust effect of condition, *F*(2, 28) = 83.52, *p* < .001, η^2^ p = 0.86. AMD was the most precise (*M* = 407, *SD* = 0.75), compared to AD (*M* = 662, *SD* = 92) and AM (*M* = 676, *SD* = 78). Post hoc comparisons confirmed that AMD differed significantly from both AD, *t*(14) = 10.88, *p* < 0.01, *d* = 3.12, and AM, *t*(14) = 11.48, *p* < 0.01, *d* = 3.29. AD and AM did not differ, *t*(14) = − 0.60, *p* = 1.00, (Fig. 2d).

In the FAST session, AMD produced the largest bias but also the highest precision, while AM yielded the smallest bias. In the SLOW session, the pattern reversed: AMD showed the lowest bias and highest precision compared to single-cue conditions. Across both temporal regimes, AMD consistently improved precision, but its effect on bias depended on TTC length—exacerbating error for short intervals but reducing it for extended intervals.

### Flat-prior Bayesian cue-combination benchmark

**Fig. 3:**
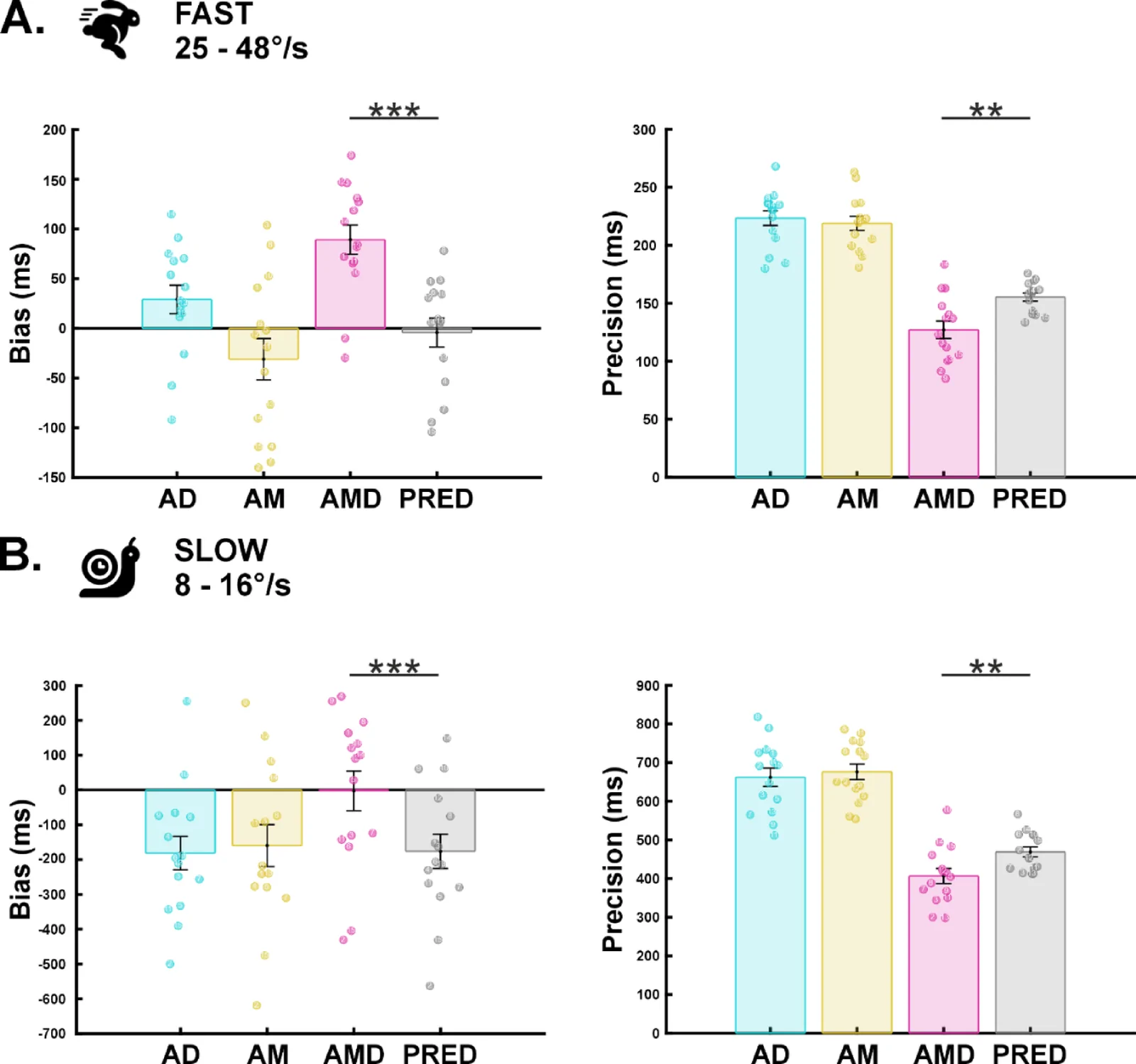
Flat-prior Bayesian cue-combination benchmark. (**A**) FAST (24–48°/s) and (**B**) SLOW (8–16°/s) conditions. Bar plots show participants’ mean bias (left) and precision (right) for the auditory duration (AD), auditory motion (AM), and combined motion-duration (AMD) conditions, along with predictions from an inverse-variance weighted benchmark (PRED). Each circle represents one participant, bars indicate group means, and error bars denote the standard error of the mean. Paired-samples t-tests compared observed AMD performance with the flat-prior benchmark predictions.(**p < 0.1, ***p < 0.01).

Observed AMD performance was compared with predictions from the flat-prior Bayesian cue-combination benchmark. In the FAST condition, observed AMD bias differed significantly from the benchmark prediction (*M* = -4, *SD* = 56), *t*(14) = 7.36, *p* < 0.01, *d* = 1.90, indicating that the flat-prior benchmark did not fully capture the systematic response bias observed in AMD. Observed AMD precision also differed from the benchmark prediction (*M* = 155, *SD* = 13), *t*(14) = -3.77, *p* = .0.02, *d* = -0.97 (Fig. 3).

In the SLOW condition, observed AMD bias and precision also differed significantly from the flat-prior benchmark predictions (Bias prediction: *M* = -176, *SD* = 191; Precision Prediction: *M* = 469, *SD* = 50) with bias: *t*(14) = 4.57, *p* < 0.01, *d* = 1.9; and with precision *t*(14) = -3.92, *p* = 0.02, *d* = -1.01.

Together, these results indicate that the flat-prior Bayesian cue-combination benchmark provided a useful reliability-weighted reference, but did not fully account for observed AMD performance across temporal contexts. This suggests that auditory TTC estimates cannot be fully explained by a simple inverse-variance-weighted average of the single-cue conditions, particularly when temporal context and systematic bias are taken into account.

### Regression-to-the-mean analysis

We ran linear regressions of estimated TTC on real TTC to quantify regression-to-the-mean effects across conditions and temporal contexts.

**Fig. 4:**
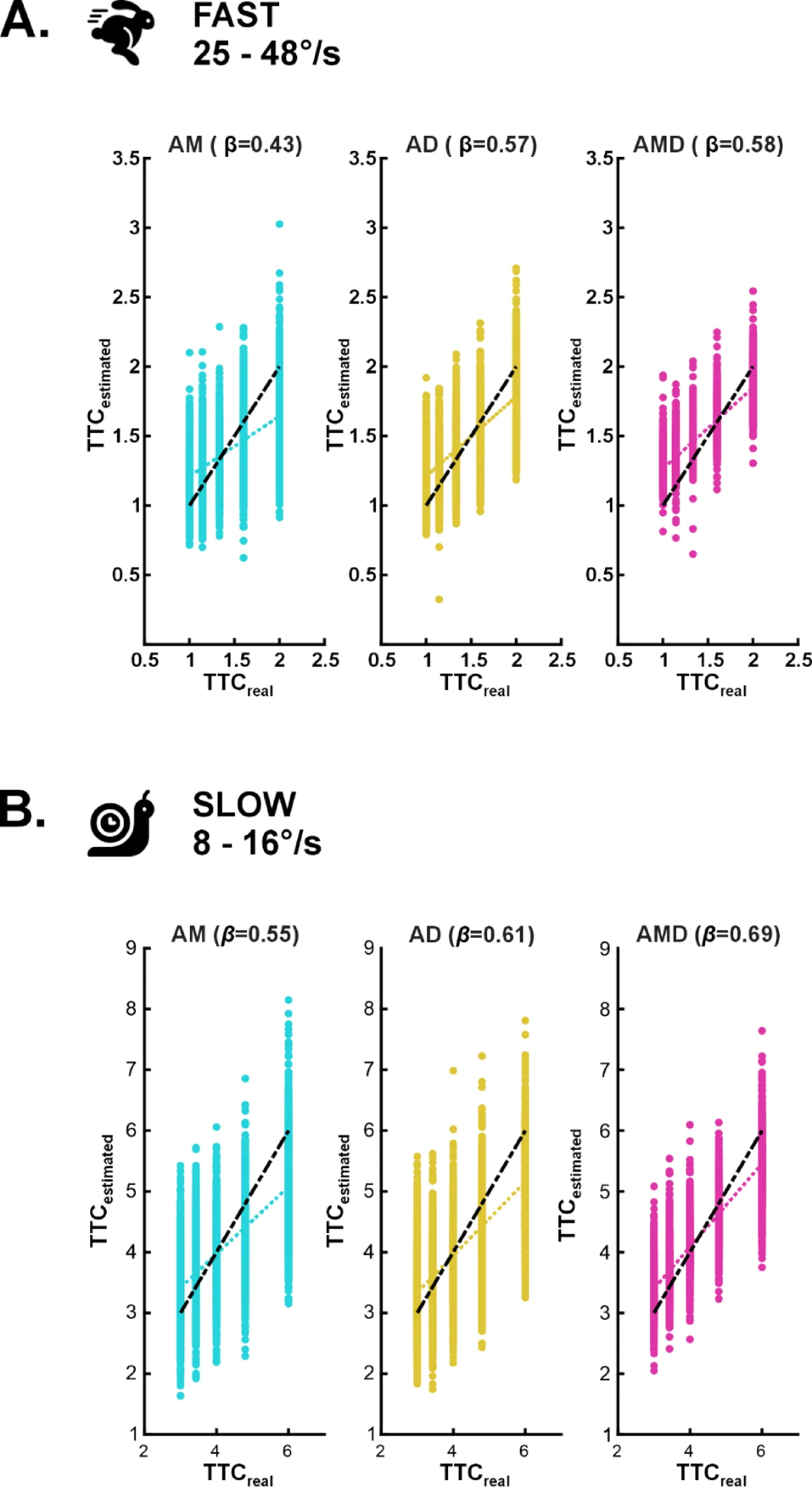
Regression-to-the-mean analysis. (**A**) FAST (24–48°/s) and (**B**) SLOW (8–16°/s) conditions. Regression-to-the-mean analysis. Scatter plots show the relationship between estimated TTC and real TTC for the auditory motion (AM), auditory duration (AD), and combined motion-duration (AMD) conditions in the FAST and SLOW temporal contexts. Coloured dotted lines indicate the best-fitting linear regressions, whereas the dashed black line represents perfect performance (slope = 1). Slopes significantly below unity indicate compression toward the mean TTC, reflecting overestimation of shorter TTCs and underestimation of longer TTCs.

Regression-to-the-Mean effects were observed in all conditions (Fig. 4). In the FAST context, slopes were significantly below unity for AM (β = 0.432, SE = 0.011, R² = 0.291, *p* < 0.01), AD (β = 0.573, SE = 0.011, R² = 0.425, *p* < 0.01), and AMD (β = 0.583, SE = 0.007, R² = 0.674, *p* < 0.01). In the SLOW context, slopes were also significantly below unity for AM (β = 0.553, SE = 0.011, R² = 0.399, *p* < .001), AD (β = 0.608, SE = 0.011, R² = 0.456, *p* < 0.01), and AMD (β = 0.685, SE = 0.008, R² = 0.695, *p* < 0.01). Here, p-values refer to one-tailed tests against unity, assessing whether each slope was significantly lower than 1. These results indicate compression toward the mean TTC, reflecting overestimation of shorter TTCs and underestimation of longer TTCs.

**Fig. 5:**
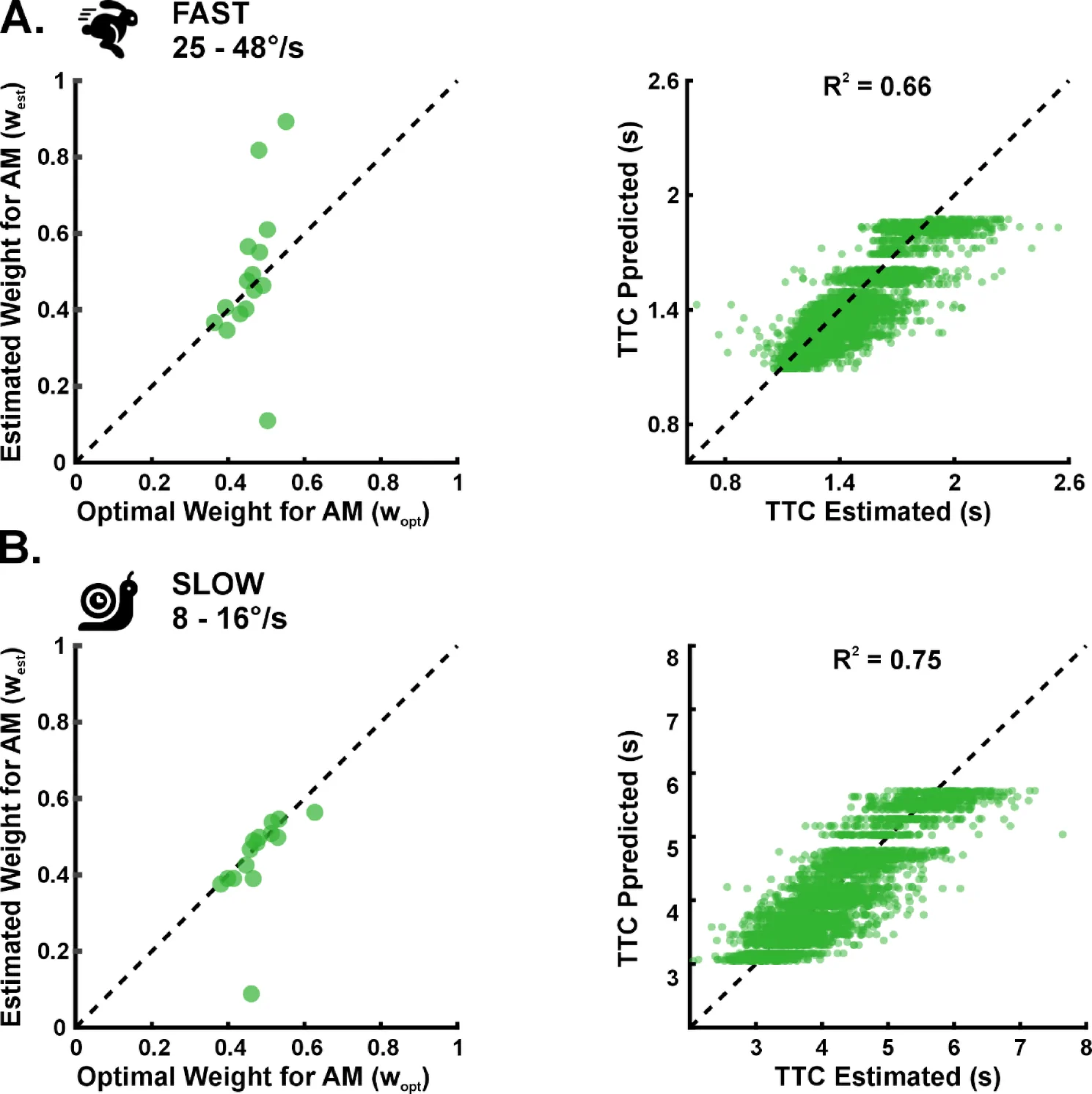
Hierarchical Bayesian model validation. (**A**) FAST (24–48°/s) and (**B**) SLOW (8–16°/s) conditions. The left panels show the relationship between the estimated weights for auditory motion (w_est_) derived from AMD responses, and the reliability-based optimal weights, w_est_, computed from the model-estimated sensory uncertainties σ_AM_ and σ_AD_ using the inverse-variance rule. The dashed line indicates perfect correspondence. The right panels display the relationship between the predicted and observed time-to-contact (TTC) values for each condition, with the proportion of explained variance (R²) reported in each plot. Together, these results indicate that the hierarchical Bayesian model accurately captures participants’ cue weighting and overall behavioral performance across motion speeds.

Overall, these findings reveal robust regression-to-the-mean effects across all conditions. However, this compression was strongest in the AM condition and was reduced when duration information was available, particularly in the AMD condition. This pattern suggests that auditory TTC judgments are influenced by expectations shaped by task statistics, but that combined motion-duration information may partially reduce this contextual bias (Figs. 4, 5).

### Hierarchical Bayesian model

Posterior estimates converged adequately in both sessions. For the FAST session, the median adequate sample size (ESS) was 83.9 (range = 14.2–205.7), with 61% of traces showing |Geweke z| < 2. For the SLOW session, the median ESS was 68.7 (range = 13.9–197.5), with 83% of traces below this threshold. Posterior means (M) and standard deviations (SD) across participants in the FAST session were as follows: σ_AM_ (*M* = 0.233, *SD* = 0.031), σ_AD_ (*M* = 0.214, *SD* = 0.024), σ_prior_ (*M* = 0.220, *SD* = 0.055), µ_prior_ (*M* = 1.495, *SD* = 0.108), w_AM_ (*M* = 0.490, *SD* = 0.187), and w_AM_^optimal^ (*M* = 0.458, *SD* = 0.048). In the SLOW session, the corresponding estimates were: σ_AM_ (*M* = 0.664, *SD* = 0.056), σ_AD_ (*M* = 0.641, *SD* = 0.096), σ_prior_ (*M* = 0.768, *SD* = 0.184), µ_prior_ (*M* = 4.026, *SD* = 0.481), w_AM_ (*M* = 0.501, *SD* = 0.118), and w_AM_^optimal^ (*M* = 0.479, *SD* = 0.061).

Comparisons of cue reliability indicated that motion uncertainty was significantly greater than duration uncertainty in the FAST session, *t*(14) = 3.26, *p* = 0.06, *d*_z_ = 0.84, 95% CI [0.0066, 0.0319]. In contrast, no reliable difference was observed in the SLOW session, *t*(14) = 1.09, *p* = 2.93, *dz* = 0.28, 95% CI [− 0.0221, 0.0699].

Observed cue weights were compared against the reliability-based benchmark $\:{w}_{optimal}$, computed from the model-estimated sensory uncertainties σ_AM_ and σ_AD_. In the FAST session, the correlation between observed and optimal weights was not significant, *r* = .30, *p* = 2.77, and the paired-samples comparison showed no difference, *t*(14) = 0.69, *p* = 5.02, *d*_z_ = 0.18, 95% CI [− 0.067, 0.131]. In the SLOW session, the correlation was more substantial and significant, *r* = 5.6, *p* = 0.31, although the paired-samples comparison again showed no difference, *t*(14) = 0.72, *p* = 4.82, *d*_z_ = 0.19, 95% CI [− 0.041, 0.084].

Model validation indicated that predictions explained 65.3% of the variance in multicue estimates during the FAST session and 74.8% during the SLOW session (Fig. 5). Exploratory analyses showed no reliable associations between w_AM and stimulus velocity (FAST: *r* = 0.2, *p* = 9.52; SLOW: *r* = − 0.3, *p* = 9.32) or between w_AM and absolute error in AMD (FAST: ρ = 0.09, *p* = 7.63; SLOW: ρ = 0.10, *p* = 0.713).

Taken together, these findings indicate that participants relied on prior information and combined motion-derived and duration-based information in a manner broadly consistent with reliability-based cue weighting. Motion cues were less reliable than duration cues in the FAST session, whereas in the SLOW session, the two cues showed comparable levels of uncertainty. Thus, the SLOW pattern should be interpreted as reflecting more balanced cue use rather than dominance of the duration cue.

## Discussion

We investigated whether auditory TTC estimation involves Bayesian integration of temporal and kinematic cues, and whether this integration is modulated by temporal context. We hypothesized that participants would integrate temporal and motion cues to optimize TTC estimation, and that this integration would be modulated by temporal context, particularly by whether events occurred in a fast or slow temporal context. We further predicted that their performance would follow Bayesian reliability-weighted integration, while also reflecting internally induced priors derived from the statistics of recent experience.

Consistent with these hypotheses, integration consistently improved precision compared to single cues. Across both fast and slow sessions, combining duration and motion (AMD) enhanced reliability beyond what could be achieved with either cue alone, confirming that auditory TTC estimation adheres to reliability-weighted cue combination^24,7^. However, temporal context played a critical role: integration introduced systematic delays in the fast session, while in the slow session, it reduced the anticipatory biases observed with single cues. This dissociation shows that integration is not a fixed computation, but an adaptive process where the outcome depends on the temporal context and the relative reliability of the cues.

The flat-prior Bayesian cue-combination benchmark provided a useful reliability-weighted reference for evaluating AMD performance^24,7^. Because the AM and AD precision estimates were derived from behavior, this benchmark should be interpreted as a descriptive reference based on observed single-cue reliability, rather than as a pure sensory-likelihood model. Deviations between observed AMD performance and the benchmark, therefore, suggest that a simple inverse-variance weighted cue-combination rule under a flat prior does not fully capture auditory TTC behavior, particularly when temporal context and regression-to-the-mean effects are considered^9,20^. Similarly, because the cue weight in the hierarchical model was freely estimated, this model should be interpreted as characterizing empirical cue-weighting behavior relative to a reliability-based benchmark, rather than as a formal comparison between optimal and suboptimal integration models.

Importantly, the observed FAST–SLOW asymmetry should not be interpreted merely as a contextual modulation, but as a shift between distinct reliability frameworks. In the FAST condition, faster auditory motion and shorter sampling windows likely made motion-derived TTC estimates relatively less reliable, leading to lower relative weighting of motion information and a stronger influence of duration-based information and contextual priors. On the other hand, in the SLOW condition, extended durations allow for more accurate temporal accumulation and better estimation of elapsed time. Under this context, in the SLOW condition, longer durations were associated with comparable motion- and duration-cue uncertainty. This pattern suggests that the two information sources may have contributed more similarly to TTC estimation, stabilizing responses without requiring a dominance of the duration cue. This interpretation aligns with Bayesian cue combination principles^24,7^ and with visual findings showing that precision gains depend on the relative reliability of speed and time information^9^. These findings are consistent with Bayesian accounts in which perceptual estimates are shaped by sensory uncertainty and prior expectations^11^. However, because FAST and SLOW trials were presented in separate sessions, the present design supports conclusions about session-level temporal-context calibration rather than trial-by-trial prior updating^21,20^.

Regression-to-the-mean analyses confirmed that internally induced priors influence auditory TTC estimation, specifically the subjective expectations formed from the range of TTCs encountered during the task. These priors stabilize perception under uncertainty but compress estimates toward the mean interval, producing systematic distortions in fast contexts when motion noise dominates. The asymmetry between temporal contexts thus reflects the interaction between cue reliability and prior-driven regularization.

Because µ_prior_ and σ_prior_ were freely estimated, they should be interpreted as model-based approximations of participants’ subjective temporal context, rather than as evidence that participants represented the objective stimulus distribution exactly. Future work could directly compare subjective-prior models with models anchored to the experimentally defined prior distribution.

This pattern also reveals a key asymmetry between modalities. In vision, TTC estimation balances spatial and temporal cues^9,3^, whereas audition prioritizes temporal anchoring, consistent with auditory specializations for duration and rhythm^27–29^. Such temporal prioritization may explain why context exerts a more substantial influence on auditory TTC biases. The delayed responses observed in fast integration (≈ 93 ms) resemble predictive illusions such as the flash-lag effect^30,31^, suggesting that predictive coding mechanisms operate across modalities, trading accuracy for perceptual stability.

A limitation of the present study is the sample size. Although the sample was sufficient to detect the main within-subject behavioral effect and is comparable to previous psychophysical TTC studies using Bayesian modeling approaches, this does not necessarily ensure highly stable estimation of participant-level parameters in the hierarchical Bayesian model. Accordingly, model-derived estimates of cue uncertainty, subjective priors, and cue-weighting should be interpreted as informative but preliminary. The model is used here mainly to provide a descriptive computational account of the observed behavioral pattern, rather than definitive estimates of individual computational parameters. Larger samples will be needed to assess the stability and generalizability of these model-based parameters.

Beyond their theoretical implications, these findings may inform future work on auditory TTC estimation and auditory warning signals. However, because participants maintained central fixation and a fixed head position, the present results should be interpreted as evidence from a controlled auditory TTC task rather than as directly generalizable to natural navigation or driving. Future studies should test whether similar temporal-context effects emerge in more ecological settings involving head movements, visual information, and dynamic attentional demands.

This study shows that auditory TTC judgments provide a critical test of Bayesian inference in perception. Overall, these results support our initial hypothesis that auditory TTC judgments arise from the integration of temporal and kinematic cues within a Bayesian framework, flexibly weighted by context and experience. Rather than reflecting a rigid computation, auditory TTC estimation emerges as a dynamic inference process shaped by internal priors and temporal structure. Indeed, participants flexibly integrate temporal and kinematic cues, relying more on duration when motion is noisy, and adjust their strategies with temporal context. These findings refine our understanding of perceptual inference, demonstrating that optimality is not a fixed ceiling but a flexible framework dynamically shaped by priors, perceptual history, and modality-specific constraints^10,32,11^. This work advances a unified view of temporal perception, linking a fundamental perceptual ability to broader predictive coding theories.

## Data Availability

Data availabilityThe dataset reported on in this study has been deposited at Zenodo at https://doi.org/10.5281/zenodo.17776516. Code availabilityCustom MATLAB scripts necessary to reproduce the flat-prior cue-combination benchmark and the hierarchical Bayesian observer model reported in the manuscript are available in the Zenodo repository: https://doi.org/10.5281/zenodo.17776516. The repository includes the essential analysis scripts and a README file describing the required cleaned input dataset and output files.

## Code availability

Custom MATLAB scripts necessary to reproduce the flat-prior cue-combination benchmark and the hierarchical Bayesian observer model reported in the manuscript are available in the Zenodo repository: https://doi.org/10.5281/zenodo.17776516. The repository includes the essential analysis scripts and a README file describing the required cleaned input dataset and output files.
